# Are normal-weight adolescents satisfied with their weight? Adolescentes eutróficos estão satisfeitos com o seu peso?

**DOI:** 10.1590/1516-3180.2015.01850912

**Published:** 2016-05-13

**Authors:** Mariana Contiero San Martini, Daniela de Assumpção, Marilisa Berti de Azevedo Barros, Ana Maria Canesqui, Antonio de Azevedo Barros

**Affiliations:** I MSc. Researcher, Department of Pediatrics, School of Medical Sciences, Universidade Estadual de Campinas (Unicamp), Campinas, SP, Brazil.; II PhD. Postdoctoral Researcher, Universidade Federal de São Paulo (Unifesp), São Paulo, and Researcher, Department of Public Health, School of Medical Sciences, Universidade Estadual de Campinas (Unicamp), Campinas, SP, Brazil.; III PhD. Titular Professor, Department of Public Health, School of Medical Sciences, Universidade Estadual de Campinas (Unicamp), Campinas, SP, Brazil.; IV PhD. Collaborating Associate Professor, Department of Public Health, School of Medical Sciences, Universidade Estadual de Campinas (Unicamp), Campinas, SP, Brazil.; V PhD. Titular Professor, Department of Pediatrics, School of Medical Sciences, Universidade Estadual de Campinas (Unicamp), Campinas, SP, Brazil.

**Keywords:** Adolescent, Adolescent behavior, Body weight, Body image, Health surveys

## Abstract

**CONTEXT AND OBJECTIVE:**

The high prevalence of obesity has led to public policies for combating it. People with normal weight may gain greater awareness of this issue and change their perceptions of their weight. The aim of this study was to evaluate the prevalence of body weight dissatisfaction among normal-weight adolescents, according to demographic and socioeconomic variables, health-related behavior and morbidities.

**DESIGN AND SETTING:**

Population-based cross-sectional study that used data from a health survey conducted in the city of Campinas, São Paulo, in 2008-2009.

**METHODS:**

The prevalence and prevalence ratios of weight dissatisfaction were estimated according to independent variables, by means of simple and multiple Poisson regression.

**RESULTS:**

573 normal-weight adolescents aged 10 to 19 years (mean age 14.7 years) were analyzed. The prevalence of weight dissatisfaction was 43.7% (95% confidence interval, CI: 37.8-49.8). Higher prevalences of weight dissatisfaction were observed among females, individuals aged 15 to 19 years, those whose households had eight or more domestic appliances, former smokers, individuals who reported alcohol intake and those who had one or more chronic diseases. Lower prevalence of dissatisfaction was observed among adolescents living in substandard housing. Among the normal-weight adolescents, 26.1% wished to lose weight and 17.6% wished to gain weight.

**CONCLUSION:**

The results from this study indicate that even when weight is seen to be within the normal range, a high proportion of adolescents express dissatisfaction with their weight, especially females, older adolescents and those of higher socioeconomic level.

## RESUMO

**CONTEXTO E OBJETIVO:**

Com a alta prevalência da obesidade levando a políticas públicas para o seu enfrentamento, pessoas com o peso adequado podem se sensibilizar e alterar sua percepção sobre o seu peso. Objetivou-se avaliar a prevalência de insatisfação com o peso corporal em adolescentes eutróficos, segundo variáveis demográficas e socioeconômicas, comportamentos relacionados à saúde e morbidades.

**TIPO DE ESTUDO E LOCAL:**

Estudo transversal de base populacional que utilizou dados do Inquérito de Saúde realizado em Campinas (SP), em 2008/2009.

**MÉTODOS:**

Foram estimadas as prevalências e as razões de prevalência de insatisfação com o peso segundo as variáveis independentes, por meio de regressão simples e múltipla de Poisson.

**RESULTADOS:**

Analisaram-se 573 adolescentes eutróficos de 10 a 19 anos, com idade média de 14,7 anos. A insatisfação com o peso atingiu prevalência de 43,7% (intervalo de confiança de 95%, IC: 37,8-49,8). Maiores prevalências de insatisfação com o peso foram verificadas no sexo feminino, nos adolescentes de 15 a 19 anos, que possuíam oito ou mais equipamentos na residência, nos ex-fumantes, nos que relataram ingerir bebida alcoólica e nos que apresentavam uma ou mais doenças crônicas. Menor prevalência de insatisfação foi observada nos adolescentes que residiam em moradias com condições inadequadas. Dos adolescentes eutróficos, 26,1% queriam perder peso e 17,6% desejavam ganhar.

**CONCLUSÕES:**

Os resultados apontam que, mesmo apresentando o peso dentro da faixa de normalidade, elevada proporção de adolescentes manifesta-se insatisfeita com o seu peso, em especial os indivíduos de sexo feminino, os mais velhos e os de melhor nível socioeconômico.

## INTRODUCTION

Obesity is a major public health problem that contributes towards the development of other morbidities, such as cardiovascular diseases, hypertension and diabetes mellitus, thus impairing quality of life and increasing the risk of mortality.^1^^,^^2^^,^^3^ The increasing prevalence of overweight raises concern regarding healthy eating and regular physical activity,^4^^,^^5^ within a context permeated by the demand for an ideal body and slimness, especially among young people.^6^^,^^7^ Paradoxically, advertising broadcast by the mass media encourages intake of foods of high energy density and low nutrient density, such as cookies, chips/crisps and fast-food snacks.^8^^,^^9^^,^^10^

Adolescence is marked by deep biological, cognitive and psychosocial changes and the experiences at this stage of life, such as the beginning of sexual activity, possible introduction of risky health-related behavior, expansion of autonomy from the family^11^^,^^12^ and greater exposure to the media^6^ may influence self-assessment of body image, possibly causing weight dissatisfaction and harmful health-related behavior.^13^

The ideal body varies according to sex among young people. Boys tend to have less concern about appearance, but desire an athletic body,^14^^,^^15^^,^^16^ while girls want a leaner body.^15^^,^^16^^,^^17^ Girls more frequently engage in diets and exercise to lose weight, may be more influenced by advertisements for weight loss products and believe that slim people are more popular and attractive.^18^ The female susceptibility to influences relating to physical appearance leads to higher risk of developing eating disorders.^18^

A study on adolescents between 14 and 19 years of age in the city of São Paulo found a distortion between the nutritional status and self-perceived body image, according to sex. Among normal-weight adolescents, 38.8% of the girls and 19.2% of the boys saw themselves as overweight, 47.6% of the girls saw themselves as obese and 26.3% of the boys saw themselves as having normal weight.^19^

Weight satisfaction is an essential factor for self-acceptance among young people, and when current weight is not compatible with the desired body, this can trigger inappropriate attitudes, thereby affecting these individuals’ growth and development.

## OBJECTIVE

Considering the increasing prevalence of obesity and its health implications, along with the fact that correct self-perception of nutritional status is essential for adopting appropriate health promotion practices, the objective of this study was to evaluate the prevalence of dissatisfaction with body weight among normal-weight adolescents living in the city of Campinas, São Paulo, Brazil, according to demographic and socioeconomic variables, health-related behavior and morbidities.

## METHODS

This was a cross-sectional population-based prevalence study that used data from a health survey conducted in the city of Campinas (ISACamp, 2008) by the Collaborating Center on Health Situation Analysis of the Department of Public Health, School of Medical Sciences, State University of Campinas (Faculdade de Ciências Médicas da Universidade Estadual de Campinas, FCM/Unicamp). Data were gathered between the months of February 2008 and April 2009.

The survey sample was determined by means of probability sampling procedures in two cluster stages: census tracts and households. In the first stage, 50 census tracts were drawn with probabilities proportional to size (number of households). The census tracts of the Brazilian Institute for Geography and Statistics (Instituto Brasileiro de Geografia e Estatística, IBGE) were used, based on the demographic census of 2000. Taking into account the time that had elapsed, the household data of the 50 census tracts selected were updated. In the second stage, households were drawn.

The survey population comprised adolescents of 10 to 19 years. The sample size was defined as 1,000 individuals for each age domain, which allowed for an estimated prevalence of 50%, with a 95% confidence level and a sampling error of between 4 and 5 percentage points, considering a design effect of 2.

By taking the response rate to be 80%, the sample size was set at 1,250. To achieve the desired sample size, 2,150 households were independently drawn for interviews with adolescents.

Information was gathered through a questionnaire structured into 14 thematic blocks. The questionnaire had been tested in pilot studies, and it was applied by trained interviewers overseen by the researchers. The thematic block relating to dietary habits contained questions on self-reported weight and height, weight satisfaction and practices used for weight loss, among others.

In the present study, teenagers aged 10 to 19 years, of both sexes, who were not institutionalized and were living in the urban area of the city of Campinas were studied. The dependent variable was dissatisfaction with body weight, which was evaluated according to responses to the question: “Would you like to gain or lose weight?” If the respondents answered yes, they were asked how much they would like to weigh. If their desire was to lose weight, they were asked whether they were doing anything to lose weight, and if so, what practices they were using for weight loss.

The independent variables analyzed in this study were as follows.

Demographic and socioeconomic factors: sex, age (in years), self-reported race/skin color, monthly per capita household income (expressed as multiples of the minimum monthly wage), number of appliances in the household, occupational activity, education level of household head (in years of schooling), possession of health insurance, school attendance (and whether the school was public or private), and housing conditions, categorized as adequate or inadequate (substandard). Housing was considered adequate when houses or apartments had an internal water supply network connected to the public system, internal sanitary installation connected to the public sewer system and electric lighting. In the absence of one or more of these conditions, the housing was characterized as inadequate.

Health-related behavior: dentist appointment within the past year, smoking status, alcohol intake, length of time exposed to a computer (hours per day) and leisure physical activity. Individuals were classified as active, if they were doing at least 60 minutes of physical activity every day on at least five days a week (individuals aged 10 to 17 years) or at least 150 minutes a week, distributed across a minimum of three days (individuals aged 18-19 years). They were classified as insufficiently active if they did physical activity below the levels above; or as inactive if they did not do any kind of recreational physical activity.^20^

Morbidities: self-reported number of chronic diseases among those included in the survey checklist, such as hypertension, diabetes or asthma/bronchitis; and number of health complaints reported in a different checklist, such as headaches/migraines, allergies or emotional problems, among others.

Nutritional status was assessed from the body mass index (BMI) [weight (kg)/height (m^2^)], which was calculated using the reported height and weight information. The adolescents’ nutritional status was classified according to the BMI cutoff points for age that are recommended by the World Health Organization:^21^ underweight BMI < 3^rd^ percentile; normal weight BMI ≥ 3^rd^ percentile and ≤ 85^th^ percentile; overweight BMI > 85^th^ percentile and ≤ 97^th^ percentile; and obese BMI > 97^th^ percentile.

The prevalence of body weight dissatisfaction among normal-weight individuals was estimated according to the independent variables. The association was verified using the χ² test, taking the significance level to be 5%. The prevalence rates and 95% confidence intervals were calculated through simple Poisson regression. The multiple model using multiple Poisson regression was developed in two stages. In the first stage, the demographic and socioeconomic variables that presented P < 0.20 in bivariate analysis were introduced, and variables with P < 0.05 were kept in the model. In the second stage, health-related behavior and morbidity variables were added to the model when any category presented P < 0.20 in bivariate analysis and were kept in the model if P < 0.05 in one of the categories.

The data were entered using Epidata 3.1 (Epidata Association, Odense, Denmark) and statistical analyses were conducted in the svy module of the Stata 11.0 software (Stata Corp., College Station, Texas, United States), which enables analysis on data from complex samples.

This study was approved by the Research Ethics Committee of the School of Medical Sciences, State University of Campinas, under Certificate of Presentation for Ethics Assessment no. 39756314.6.0000.5404.

## RESULTS

In this study, only normal-weight adolescents were analyzed, totaling a sample of 573 individuals, with an average age of 14.7 years (95% confidence interval, CI: 14.5-14.9). Therefore, out of the sample of 822 individuals with BMI assessments, 249 were excluded because they were underweight, overweight or obese.

It was observed that 56.2% (95% CI: 50.2-62.1) of the adolescents with appropriate nutritional status were satisfied with their body weight. Among those who were dissatisfied with their weight, 17.6% reported a desire to gain weight, 18.0% wished to lose less than 10% of their weight and 8.1% wished to lose 10% or more. Among the boys, 65.5% said that they were satisfied with their weight, 19.8% wanted to gain weight, 9.9% wanted to lose < 10% and 4.8% wanted to lose ≥ 10%. Among the girls, 48.0% were satisfied with their weight, 15.6% wanted to gain weight, 25.3% wanted to lose < 10% and 11.1% wanted to lose ≥ 10% of their current weight.

In Table 1, higher prevalence of weight dissatisfaction can be observed among females, individuals between the ages of 15 to 19 years, those with eight or more appliances in the home and those who did not attend school. On the other hand, those who reported not working and those living in substandard housing showed significantly lower prevalence of weight dissatisfaction.

**Table 1.**
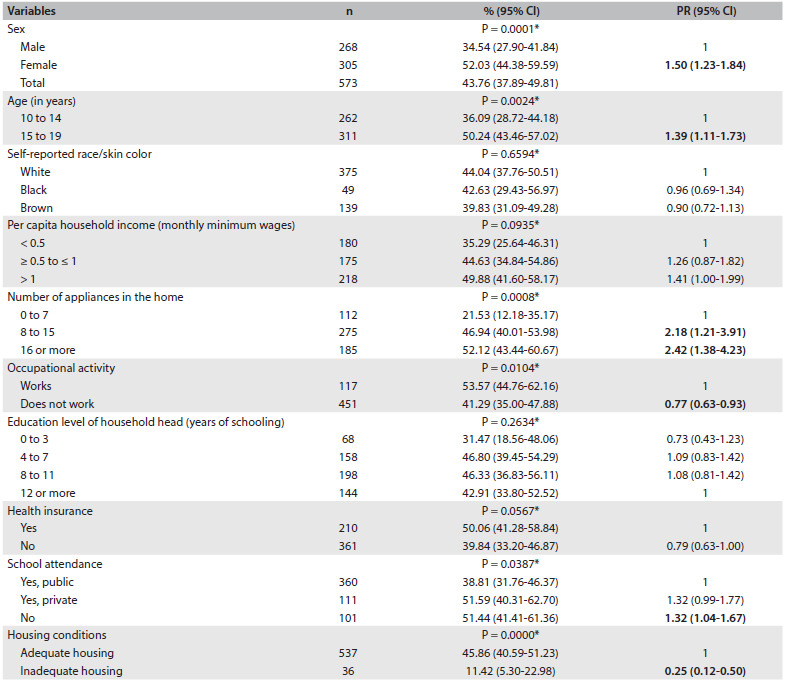
Prevalence of body weight dissatisfaction among normal-weight adolescents aged 10 to 19 years, according to demographic and socioeconomic variables. Health Survey of Campinas (ISACamp, 2008/2009)

n = number of individuals in the unweighted sample; 95% CI = 95% confidence interval; PR = prevalence ratio. *P-value from chi-square test.

Regarding health-related behavior and morbidities (Table 2), greater prevalence of weight dissatisfaction was seen among the adolescents who were former smokers, those who drank alcohol, those who used computers for three or more hours/day, those who reported having one or more chronic diseases and those who reported having three or more health complaints.

**Table 2.**
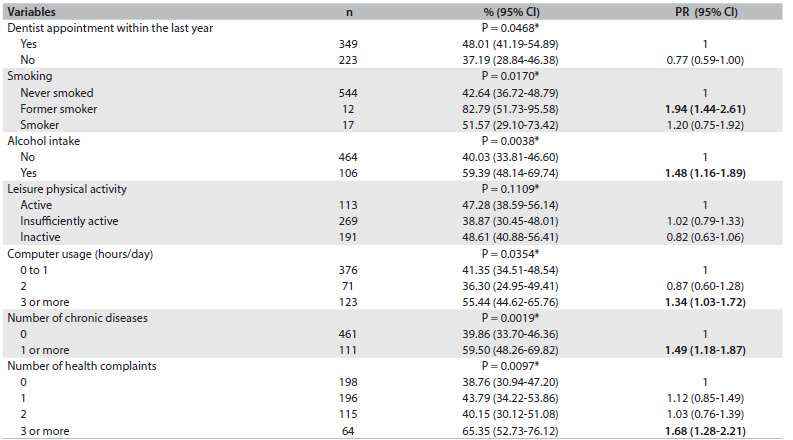
Prevalence of body weight dissatisfaction among normal-weight adolescents aged 10 to 19 years, according to health-related behavior and morbidity variables. Health Survey of Campinas (ISACamp, 2008/2009)

n = number of individuals in the unweighted sample; 95% CI = 95% confidence interval; PR = prevalence ratio. *P-value from chi-square test.

The results from the multiple Poisson regression (Table 3) revealed that there was higher prevalence of weight dissatisfaction in the age group of 15 to 19 years, among females and among individuals who belonged to the category with the greatest number of appliances in the home. Living in substandard housing was associated with lower prevalence of weight dissatisfaction. Former smokers, individuals who drank alcoholic beverages and those with one or more chronic diseases were more dissatisfied with their current weight.

**Table 3.**
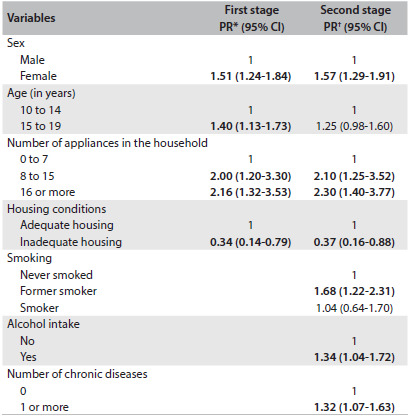
Poisson multiple regression model in two stages. Health Survey of Campinas (ISACamp, 2008/2009)

95% CI = 95% confidence interval. *Prevalence ratio adjusted for demographic and socioeconomic variables; ^†^Prevalence ratio adjusted for all variables in the table.

Table 4 presents the prevalence of weight satisfaction and the wish to gain or lose weight, among adolescents with healthy weight. The highest rates of desire to lose weight were seen among female adolescents, among females in the 15-19 year age group and among individuals living in homes with eight or more appliances. The prevalence of the desire to gain weight was greater among males between 15 and 19 years of age and among individuals living in homes with eight or more appliances.

**Table 4.**
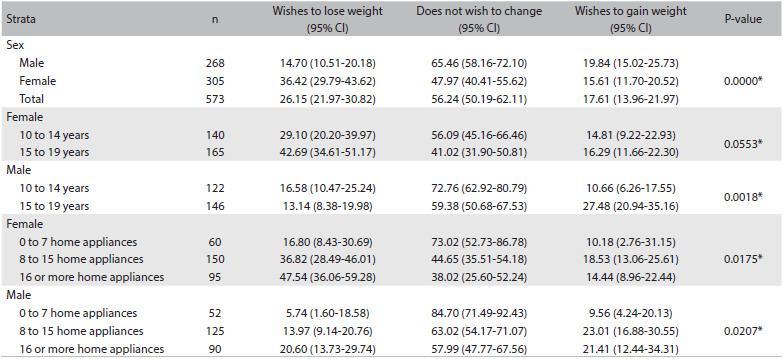
Distribution of desire to change weight among normal-weight adolescents, according to sociodemographic strata

95% CI = 95% confidence interval; n: number of subjects in unweighted sample. *P-value from chi-square test.

## DISCUSSION

This study demonstrated that despite these adolescents’ appropriate weight, they manifested high prevalence of weight dissatisfaction, especially females, individuals between the ages of 15 and 19 years, those of higher socioeconomic level (as assessed according to the number of household appliances and adequacy of housing conditions), former smokers, individuals who were consuming alcohol and those who presented at least one chronic disease. Among the normal-weight adolescents, 43.7% presented weight dissatisfaction. In an analysis on a sample of 594 adolescents between the ages of 15 to 20 years who were enrolled in public schools in Caruaru, Pernambuco, Brazil, Santos et al.^22^ found that 55.1% of the adolescents within the normal weight range were dissatisfied with their weight.

Compared with the boys, the girls presented higher prevalence of weight dissatisfaction, thus corroborating the findings of other authors. In an evaluation on 17,817 Palestinians between the ages of 12 and 18 years, Al Sabbah et al.^23^ observed that 16.0% of the boys and 24.0% of the girls presenting healthy nutritional status were dissatisfied with their weight. Using silhouette scales among 4,325 individuals between the ages of 14 and 15 years, Dumith et al.^24^ found that 58.2% of the girls and 43.9% of the boys within the normal weight range presented body dissatisfaction. Among the students at a public school in the city of São Paulo, 43.6% of the girls and 19.2% of the boys with appropriate weight considered themselves to be overweight.^19^

A longitudinal study conducted in Juiz de Fora, Minas Gerais, among 358 students aged 11 to 14 years, showed that the prevalence of body dissatisfaction increased among girls as they grew up, while the opposite was observed among boys.^25^ In an evaluation on 3,096 Irish students of healthy weight, Kelly et al.^26^ found significantly greater prevalence of weight dissatisfaction among the oldest individuals, females and individuals who belonged to categories of higher socioeconomic level, as assessed through their parents’ occupations.

The present study found higher prevalence of dissatisfaction with weight both among former smokers and among individuals who consumed alcohol. Similar results were found by Xie et al.^27^ among females who smoked and drank five or more doses of alcohol on a single occasion. Among a sample of 4,746 adolescents in Minnesota, United States, Crow et al.^28^ found that there was a significantly higher proportion of alcohol intake (42.5%) and tobacco use (38.9%) among girls who dieted to lose weight, compared with those who did not do any dieting. Okeke et al.^29^ observed that adolescents who had never smoked had a more positive perception of body image. They concluded that many individuals believed that cigarettes acted as a means of weight control^29^ or weight loss and that they regulated emotions, whereas in reality body dissatisfaction had the power to trigger the start of smoking.^30^

Individuals who reported the presence of one or more chronic diseases had greater prevalence of weight dissatisfaction. A study showed that young people with chronic diseases reported greater body dissatisfaction and engagement in inappropriate weight loss practices.^31^ Another study conducted among 9,584 adults over the age of 20 years found that people who were dissatisfied with their weight, independent of BMI, presented higher risk of developing type 2 diabetes.^32^ The finding of greater dissatisfaction among adolescents with normal weight and chronic disease, requires further studies in order to understand this association.

Exposure to the media can contribute towards body dissatisfaction among adolescents between 14 and 16 years of age.^33^ Hargreaves and Tiggemann^14^ suggested that male adolescents do not express their body dissatisfaction because they believe that this is a feminine topic. On the other hand, among young women, the type of media exposure (television and magazines) can influence body dissatisfaction differently.^34^

Another communication medium that deserves attention is the internet, which is used about four times more than magazines. The internet is the only means of providing instant access to diversified content and images, while magazines depict specific and limited subjects. In the United States, similar associations regarding body dissatisfaction were found among undergraduate women exposed to television and the internet.^35^ Tiggemann and Slater^36^ found that use of social media, like Facebook, caused greater desire to lose weight, greater attention towards the body and greater internalization of the notion of slimness among female users of social media than among nonusers.

It has been highlighted that there is a tendency in contemporary society to value a beauty paradigm characterized by slim women and muscular men. The idea that this physical appearance is considered ideal and should be sought at any cost has been disseminated. Nonetheless, this may cause harm to adolescents’ health and contribute towards development of eating disorders.^19^^,^^28^^,^^37^

Brazilian studies that have taken a qualitative approach have suggested that the body can be treated as capital (physical, symbolic, economic or social). It is also an important means of access to the labor market, sexual activity, marriage and social ascension towards prestige positions, success and money. Contemporary culture recommends that the body should always be displayed as young, sexy and in good shape.^38^^,^^39^ According to Goldenberg,^38^^,^^39^ clothing is an instrument to promote and expose the body, which is displayed, shaped, produced and worked. In order to achieve the desired physical form, discipline, dedication and great investments are necessary, which can result in significant dissatisfaction with one’s appearance.^39^ Another qualitative study, carried out in Santa Catarina, pointed out that the female body should display beauty, slimness, power of attraction and seduction towards the opposite sex, while the male body should be endowed with physical strength, power and virility.^40^

Among the limitations of the present study, it is important to highlight that self-reported information was used to ascertain adolescent weight and height. This is an especially important point at this stage of life, at which there are great changes to measurements caused by rapid growth and physical development. However, several studies have indicated that there is good agreement between reported and assessed height and weight measurements among adolescents, and have therefore considered that it is valid to use this information in epidemiological studies.^41^^,^^42^^,^^43^

It is important to mention that very few studies have evaluated the factors associated with body weight dissatisfaction, especially among normal-weight individuals, even though body image and body dissatisfaction are topics greatly explored in the literature.

The results from this study draw attention to the high percentage of adolescents who do not identify their nutritional status correctly. Healthy eating and health promotion programs, along with the fight against obesity, should take this point into account.

## CONCLUSIONS

In this study, 43.8% of normal-weight adolescents were dissatisfied with their body weight. Greater prevalence of body weight dissatisfaction was found among girls, individuals aged 15 to 19 years, those at a higher socioeconomic level, former smokers, individuals who drank alcohol and those who reported having a chronic disease.
